# SAMe, Choline, and Valproic Acid as Possible Epigenetic Drugs: Their Effects in Pregnancy with a Special Emphasis on Animal Studies

**DOI:** 10.3390/ph15020192

**Published:** 2022-02-03

**Authors:** Asher Ornoy, Liza Weinstein-Fudim, Maria Becker

**Affiliations:** 1 Adelson School of Medicine, Ariel University, Ariel 40700, Israel; mariabe@ariel.ac.il; 2 Department of Medical Neurobiology, Hebrew University Hadassah Medical School, Jerusalem 9112102, Israel; liza.weinstein-f@mail.huji.ac.il

**Keywords:** S-adenosylmethionine (SAMe), valproic acid (VPA), choline, epigenetic modulators, gene expression, gestation, epigenetic diseases

## Abstract

In this review, we discuss the functions and main effects on pregnancy outcomes of three agents that have the ability to induce epigenetic modifications: valproic acid (VPA), a well-known teratogen that is a histone deacetylase inhibitor; S-adenosylmethionine (SAMe), the most effective methyl donor; and choline, an important micronutrient involved in the one methyl group cycle and in the synthesis of SAMe. Our aim was to describe the possible effects of these compounds when administered during pregnancy on the developing embryo and fetus or, if administered postnatally, their effects on the developing child. These substances are able to modify gene expression and possibly alleviate neurobehavioral changes in disturbances that have epigenetic origins, such as autism spectrum disorder (ASD), depression, Rett syndrome, and fetal alcohol spectrum disorder (FASD). Valproic acid and SAMe are antagonistic epigenetic modulators whether administered in utero or postnatally. However, VPA is a major human teratogen and, whenever possible, should not be used by pregnant women. Most currently relevant data come from experimental animal studies that aimed to explore the possibility of using these substances as epigenetic modifiers and possible therapeutic agents. In experimental animals, each of these substances was able to alleviate the severity of several well-known diseases by inducing changes in the expression of affected genes or by other yet unknown mechanisms. We believe that additional studies are needed to further explore the possibility of using these substances, and similar compounds, for the treatment of ”epigenetic human diseases”.

## 1. Introduction

With the advancement of our understanding of epigenetic mechanisms, increasingly more congenital malformations and human diseases are considered to have an epigenetic etiology [1,2,3]. Many of them affect the central nervous system and are among the leading psychiatric diseases, including depression, schizophrenia, and autism spectrum disorder [1,2,4]. Some teratogens (i.e., valproic acid) also exert damaging effects via epigenetic mechanisms [3,5]. These teratogens primarily affect the nervous system.

Long-term epigenetic changes are generally induced during developmental phases when epigenetic reprogramming occurs. The major round of epigenetic reprogramming takes place during embryonic development soon after fertilization, when most modified cytosines (5mC) are erased and de novo methylation events occur during the prenatal stage, forming the “future” epigenetic settings [6,7]. In humans, organ-specific DNA methylation patterns are highly dynamic between weeks 9 and 22 of gestation [8], during which different immature organ systems are sensitive to functional defects and malformations. Additionally, a large fraction of tissue-specific methylation patterns is laid down during the early postnatal period [9,10].

Many environmental agents that are able to interfere with normal embryonic and fetal development exert their teratogenic and embryotoxic effects by inducing epigenetic changes, interfering with gene expression, and therefore inducing typical functional and morphological deviations. For example, VPA and alcohol are both well-known neuroteratogens but can also induce a variety of congenital malformations [5,11]. These epigenetic modifiers may affect humans and various animals.

The brain seems to respond differently from many organs to epigenetic changes [1,12] possibly because major developmental events in the brain occur at different times during prenatal and early postnatal development. This long developmental period seems to widen the temporal window of vulnerability and the number of developmental processes that may be affected by epigenetic changes [9,13,14].

Many attempts to prevent some of these epigenetic modifications have been carried out, mainly in experimental animals. Attempts to prevent the damage using more physiologic “antagonistic” epigenetic modifiers have offered some success. For example, the treatment of pregnant mice exposed to large doses of alcohol with the methyl donor choline helped to alleviate the signs and symptoms induced by prenatal alcohol [15]. This led to some clinical trials with minimal success [16]. VPA-induced autistic-like behavior in mice was prevented by concomitant administration of S-adenosyl methionine [17,18].

**Figure 1:**
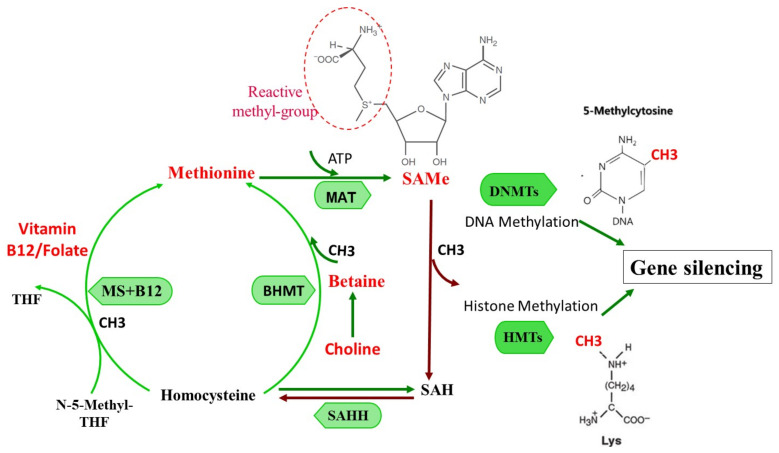
One-carbon metabolism and SAMe formation and function: Pathways of methionine, choline, and vitamin B6, B12–folate metabolism in the transmethylation cycle.

In this review, we will therefore discuss the epigenetic effects of three substances: VPA, which is a teratogen that induces histone deacetylase inhibition, thus affecting gene expression; SAMe, which is the physiologically most important methyl donor of living organisms; and choline, which is also involved in DNA methylation. Choline and SAMe are directly involved in one carbon metabolism while VPA is involved indirectly via its effects on histones. See Figure 1 for details of the one carbon cycle.

We will discuss their effects during pregnancy on the developing embryo and fetus and specifically report on the studies related to their use as epigenetic modifiers that may ameliorate damage induced by “epigenetic” teratogens.

Methionine is catalyzed by methionine adenosyltransferase (MAT) to generate S-adenosylmethionine (SAMe). SAMe donates active methyl groups to the acceptor and is converted to S-adenosylhomocysteine (SAH). SAH hydrolase (SAHH) then catalyzes the reversible hydrolysis of SAH to form homocysteine. Two pathways metabolize homocysteine to resynthesize methionine from homocysteine. In the vitamin B12/folate pathway, the reaction is catalyzed by methionine synthase (MS), which requires normal concentrations of N-5-Methyl tetrahydrofolate (N-5-Methyl-THF) and vitamin B-12. A second pathway is catalyzed by betaine-homocysteine methyltransferase (BHMT), which requires betaine, a metabolite of choline.

SAMe donates the methyl group to DNA methyltransferases (DNMTS) or histone methyltransferases (HMTs), contributing to increased genomic methylome, and to gene silencing.

## 2. Valproic Acid (VPA)

VPA has been available on the market as an anticonvulsant since 1974, and is widely used because of its efficiency against several types of epilepsy and as a mood stabilizer for the treatment of bipolar disorder and against migraine. In the last few years, it has also been used in clinical trials to treat several types of malignancies, especially advanced prostatic carcinoma [19]. Due to its known teratogenic effects, it is generally contraindicated in pregnancy unless strictly needed [5].

The use of VPA during pregnancy in humans is associated with a 1–2% incidence of neural tube defects, being associated mainly with lumbosacral meningomyelocele (spina bifida aperta) [5,20]. Other congenital malformations associated with exposure to valproic acid include facial clefts, and cardiovascular, genital (i.e., hypospadias), and limb abnormalities [21]. VPA, when added to other anticonvulsants, markedly increases the rate of malformations otherwise observed by the other drug alone. In addition, a specific “fetal valproate syndrome” has also been reported in several epidemiologic studies [5,22]. Infants with “fetal valproate syndrome” (VPA embryopathy) are prone to a variety of neurodevelopmental problems, including autism spectrum disorder (ASD). These data are based on prospective and retrospective studies of thousands of children born to VPA-treated mothers.

There seems to be a threshold regarding the teratogenic effects of VPA, ranging between daily doses of 600 and 1000 mg/day [5].

### 2.1. VPA and Autism Spectrum Disorder (ASD)

A possible association between in utero VPA exposure and ASD was described by several investigators about 30 years ago. These children often also had the typical facial features of VPA embryopathy [23]. Many reports appeared thereafter in the literature associating VPA exposure with ASD. The rate of ASD among the offspring of women treated with VPA during pregnancy was found to be 3–5 times higher compared to the general population in a large epidemiologic study [24].

Various animal studies were carried out in order to mimic the effects of VPA on the human embryo and elucidate the mechanism/s of its teratogenic action. In most animals, the drug was teratogenic, but the effective teratogenic doses differed widely. VPA induces malformations of multiple organs in rodents that resemble those described in humans, including various neurobehavioral deficits resembling ASD in mice and in rats [25,26]. Rodier et al. found [27] that the injection of 350 mg^−1^ kg^−1^ body weight to rats during days 11.5–12.5 of pregnancy reduced the number of neurons in motor cranial nerve nuclei and the size of the cerebellar hemispheres and also reduced the number of cerebellar Purkinje cells. It also caused a variety of behavioral changes mimicking ASD in human [28]. Similar changes were also observed in mice. Indeed, VPA-induced ASD-like behavior in rodents is a very popular model used to study the different aspects of human ASD [5,16,17].

The proposed mechanisms of VPA’s action on the brain include increased glutamatergic neural density, which leads to an excitatory/inhibitory imbalance, altered monoamines brain turnover, and increased reactive oxygen species [17,29]. The most important proposed mechanism is the induction of epigenetic changes due to the known VPA inhibition of histone deacetylase, possibly affecting DNA methylation [17,18,30].

Further support for an epigenetic mechanism can be found by the fact that ASD-like behavioral changes were transmitted for 3 generations in the offspring of mice treated on day 12 of gestation with a single dose of VPA [31].

### 2.2. Inhibition of Histone Deacetylases (HDACs) by VPA: The Effects on the Epigenome

The most popular proposed mechanism of VPA teratogenicity involves a combination of possible changes in gene expression resulting from histone deacetylase inhibition induced by VPA in the embryo [32,33,34,35,36,37].

**Figure 2:**
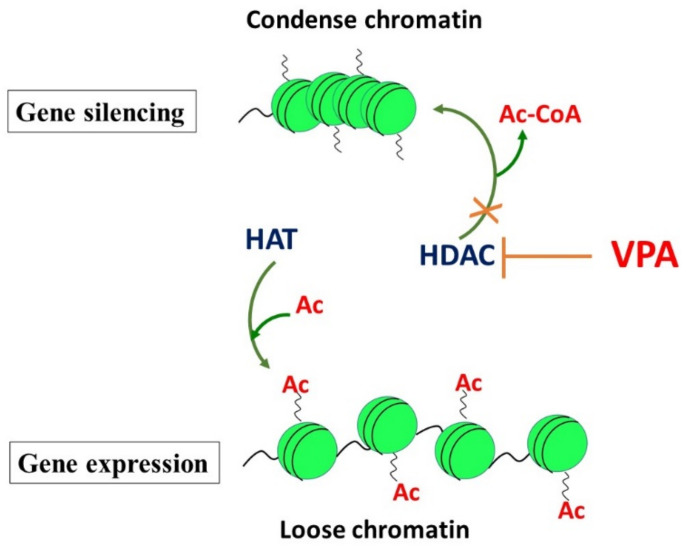
Effects of histones on gene silencing and activation and the role of VPA as a histone deacetylase inhibitor.

DNA is wrapped around the histone protein octamer, which contains two copies of histone molecules (H2A, H2B, H3, and H4) [38]. This complex nucleosome is the basic unit of chromatin. The main function of histones is the packaging of genomic DNA inside the nucleus. Nucleosomes are connected via DNA folded around the stabilizing linker histones-1 (H1). Histone modification is a dynamic process controlled by the antagonistic actions of large families of enzymes, such as histone acetyltransferases (HATs), histone deacetylases (HDACs), and methyltransferases (HMTs)/demethylases (HDMTs). Histone acetyltransferases (HATs) transfer an acetyl group from an acetyl-CoA molecule to the N-ε-lysine residues of histone, resulting in the neutralization of its positive charge and in the activation of gene transcription. In contrast, histone deacetylases (HDACs) remove the acetyl group from the N-ε-lysine residues of histone, providing a tight interaction between DNA and histone protein. This reduction of acetylation of histone 1, 3, and 4 induces chromatin changes (Figure 2). Transcription factors and RNA polymerase interact with DNA to modulate the transcription of genes. HDAC inhibitors (i.e., VPA) are able to interrupt the cell cycle, and induce growth arrest and apoptosis. Histone methylation is catalyzed by histone methyltransferases (histone-lysine N-methyltransferases and histone-arginine N-methyltransferases), which transfer one to three methyl groups from SAMe to the lysine or arginine residues of histone proteins [39].

Detich et al. [33] showed that VPA triggers active demethylation of DNA in cultured human embryonic kidney cells, and that this process is not dependent on cell replication. This active demethylation is apparently achieved by acetylation of H_3_ histones that results from the direct action of VPA as an HDAC inhibitor, increasing the accessibility of demethylases to DNA. VPA downregulated the expression of protein kinase C isoforms, inducing various changes in gene expression, such as bcl-2 and Hoxa1, and activated Wnt-dependent gene expression [34]. VPA, similar to other histone deacetylase inhibitors, such as trichostatin A and sodium butyrate, induced apoptosis in cultured microglial cells obtained from the brains of 2-day-old rat pups [37]. It also induced epigenetic changes in the decidual cells of mice treated with a single injection of VPA. This was manifested by increased levels of acetylated histone H_3_, histone H_4_, and trimethylated H3K56 [40]. In this context, it is also interesting to note that Felisbino et al. [41] added valproic acid to hepatocytes cultured in a high-glucose (hyperglycemic) medium and found that VPA decreased the expression of genes related to the complement and coagulation cascade. The attenuation of the coagulation cascade was beneficial in reversing the increased coagulopathy induced by hyperglycemia. Thus, in this context, VPA served as an epigenetic therapeutic agent.

### 2.3. Changes in Gene Expression in Offspring Induced by VPA Exposure during the Prenatal or Early Postnatal Periods

There are relatively few studies on the changes in gene expression following prenatal treatment with VPA. Most of these changes were described in the brain of rodent offspring in studies assessing VPA-induced ASD-like behavior [42,43,44,45]. Roy et al. [42] treated mice with reduced activity of methylenetetrahydrofolate reductase (Mthfr) with a single dose of 300 mg/kg VPA on day 8.5 of gestation and compared the outcome to similarly treated normal mice. While VPA induced an increased resorption rate and dead embryos in the normal mice, in the MTHFR-deficient mice, the rate of embryonic resorptions and fetal death was similar to control saline-treated mice. VPA treatment increased MTHFR gene expression, normalizing MTHFR protein synthesis in the MTHFR-deficient mice. Similar findings were observed by Guo et al. [43], who treated young Mecp2-null mice that exhibited symptoms of Rett syndrome due to MeCP2 deficiency with 350 mg/kg VPA for 2 weeks and found significant improvement in their clinical symptoms and enhanced expression of MeCP2 gene in the brain compared to non-treated mice. These studies demonstrated the ability of VPA to induce epigenetic modifications and the possibility to use VPA for the treatment of some epigenetic diseases.

Cohen et al. [44] treated pregnant rats with a single dose of 350 mg/kg VPA on day 13 of pregnancy (post major organogenesis and closure of the neural tube) and studied the gene expression in three regions of the brain in 75-day-old offspring: the amygdala, cerebellar vermis, and orbitofrontal cerebral cortex. They found significant changes in gene expression induced by VPA, with special enrichment of genes encoding acetylation-sensitive proteins, demonstrating the effects of VPA as a deacetylation inhibitor (histone deacetylase inhibitor). In addition, they found increased social investigation and play fighting in the prenatally treated rats. These behaviors increased during late adolescence. (Table 1).

Jacob et al. [45] studied the effects of 0.625 mM of VPA on zebrafish embryos and found that VPA downregulated the proneuronal gene ascl1b, causing inhibition of serotonin synthesis in the brain. These epigenetic effects of VPA on the embryonic brain were explained by the inhibition of histone deacetylase 1 by VPA, inducing a loss of serotonergic neurons.

We treated 4-day-old ICR mice with a single intraperitoneal injection of 300 mg/kg VPA to serve as a model for ASD. We were interested in studying the possible epigenetic effects of VPA by assessing gene expression in the prefrontal cortex. In addition, we investigated the possibility that these effects can be alleviated by concomitant oral administration of 300 mg/kg SAMe during postnatal days 4–6 [17,18,30]. We found that VPA treatment indeed induced ASD-like behavior (observed on postnatal days 50–60) and also induced significant changes in the expression of many genes in the brain. These changes were different between male and female mice. Co-administration of SAMe reversed most of these molecular changes induced by VPA, demonstrating the antagonistic effect of these substances (Table 1). Postnatal treatment with SAMe did not induce significant changes in gene expression.

### 2.4. In Summary

VPA is a known human and animal teratogen that induces various congenital malformations, including distinct neurobehavioral abnormalities. The main mechanism of its teratogenic action is epigenetic, as it is a potent inhibitor of histone deacetylase. In the last several years, due to its epigenetic activity, it has also been used for the treatment of several cancers [46,47,48]. The possible use of VPA as an effective epigenetic drug in pregnancy is questionable due to its high teratogenicity. However, recent attempts to find VPA derivatives that are not teratogenic have been partially successful [49,50]. Such non-tertogenic derivatives with epigenetic effects will pave the way for their use as possible epigenetic drugs in pregnancy as well. It has already been shown that VPA alleviated clinical symptoms in mice with reduced activity of the Mthfr gene and in Mecp2-deficient mice that exhibit symptoms of Rett syndrome. Additional studies are needed to evaluate the abilities of VPA to serve as a human epigenetic drug especially when used in sub-teratogenic doses.

## 3. S-adenosylmethionine: The Principal Physiologic Methyl Donor

S-adenosylmethionine (SAMe, also known as AdoMet) is a natural biological active methyl donor that is involved in most methylation reactions in all living organisms [51,52,53]. SAMe is an important cofactor for numerous metabolic processes via three main interconnected metabolic pathways: polyamine synthesis, trans-methylation, and trans-sulfuration [51,52], and is considered second only to ATP [54]. SAMe is synthesized in the cytosol of every cell, but the liver plays the central role in the homeostasis of SAMe as the major site of its synthesis and degradation [55].

SAMe has been proposed as a potential treatment for many medical conditions, particularly major depressive disorder (MDD) [56,57,58], primary and secondary fibromyalgia [56,59], attention-deficit hyperactivity disorder [60], Parkinson’s disease [61], and Alzheimer disease [62]. In addition, SAMe demonstrated good clinical effectiveness and was well tolerated in patients affected by osteoarthritis [63]. SAMe supplementation elevates hepatic glutathione (GSH) deposits and attenuates alcohol liver disease and viral cirrhosis [64,65,66,67]. Moreover, SAMe was proven in many pre-clinical studies as a potential anticancer therapeutic [68,69,70]. SAMe is an FDA-approved dietary supplement in the U.S., sold without prescriptions over the counter (OTC), but in several countries in Europe, SAMe is a prescription drug. SAMe’s oral bioavailability is near 2.1–2.6% [71] and when absorbed, SAMe enters the portal circulation and is metabolized in the liver [64].

### 3.1. SAMe as an Epigenetic Modulator

Epigenetic changes are related to a variety of congenital malformations and diseases [1,72,73]. The disruption of epigenetic machinery can lead to oxidative stress, metabolic syndrome, obesity, insulin resistance, diabetes, and vascular dysfunction in humans [74,75]. Gene expression is regulated by complex epigenetic machinery via three main mechanisms: DNA methylation, modifications of core histone proteins, and by non-coding RNAs [4,76,77].

DNA methylation is a biochemical reaction of moving the methyl group from S-adenosyl-methionine (SAMe) to the 5′-position of a cytosine nucleotide linked to a guanine nucleotide (CpG) by a phosphodiester bond, usually at CpG islands, which are located in the regulatory regions of many genes, including promoters and enhancers [78]. An altered DNA methylation state, such as hypomethylation or hypermethylation, can either facilitate or inhibit the expression of genes [79]. In the healthy mammalian genome, over 85% of CpG dinucleotides are methylated, and this methylation rate seems to be required for appropriate maintenance of chromatin integrity and transcriptional regulation [80]. The exception is imprinted genes, where the CpG islands in the promoter regions are usually unmethylated. Approximately 1% to 2% of the human genome consists of CpG dinucleotides, whose methylation is inversely related to the transcriptional activity in cells [81].

SAMe is a major methyl donor for a family of DNA methyltransferases (DNMTS), with DNMT3A/DNMT3B being responsible for de novo methylation and DNMT1 recognizing hemi-methylated DNA. An increased concentration of SAMe stimulates DNA methyltransferase reactions, launching hyper-methylation of genes [82]. This hyper-methylation state of genes protects the genome against global hypomethylation and appears as a hallmark in cancer [82], Alzheimer’s disease [83], and depression [84].

Reduced SAMe production and transmethylation pathways are associated with delayed brain development and severe neuropsychiatric diseases [85]. Elevated levels of SAMe in the peripheral nerves are characterized by DNA hypermethylation at the promoter and enhancer regions of several genes involved in lipid synthesis, leading to peripheral myelin defects [86]. In contrast, reduced SAMe levels are correlated with DNA demethylation in peripheral nerves. It was shown that a methionine-restricted diet led to reduced hepatic SAMe levels and may cause hepatocellular carcinoma (HCC) in mice [87]. In humans, reduced SAMe levels are associated with altered MAT activity and chronic liver disease (reviewed in [88]).

### 3.2. SAMe Effects on the Embryo, Fetus, and Neonate: Its Role as an Epigenetic Modulator

DNA methylation patterns and histone modifications during mammalian embryonic development undergo dynamic epigenetic processes required to establish the proper genomic epigenetic program. A first round of epigenetic reprogramming and genome-wide changes in DNA methylation occurs immediately after fertilization and during pre-implantation [7,89,90]. Massive DNA demethylation occurs between fertilization and the two-cell stage [89] and continues as the human embryo progresses from the morula stage to the blastocyst stage in the inner cell mass (ICM). After fertilization, the paternal genome is demethylated faster at the zygotic stage than the maternal genome, which is gradually demethylated during the development of the blastocyst. At the blastocyst stage, most methyl marks are removed, except elements regulating genomic imprinting and retroviral elements [80]. It was shown in the human embryo that in demethylated DNA, the promoter region of active genes associates with the presence of the trimethylation of histone H3 at the lysine 4 (H3K4me3) mark [89]. This genome-wide erasure of DNA methylation in the early embryo ensures that the genome is essentially devoid of epigenetic memory, which is important for setting up a pluripotent ground state and is probably regulated by histone modification epigenetic tools.

At the post-implantation stage, a second round of epigenetic changes takes place, and the level of methylation is sharply increased [89]. There is also an increase in the activity of the main de novo DNA methylation (DNMT3A/B) enzymes and of DNA methylation mark erasers, 10–11 translocation (TET1 and TET2) dioxygenases enzymes, with a simultaneous increase in 5hmC in the inner cell mass [91]. Failure in the function of DNMTs or TET dioxygenases may compromise normal embryonic development. The critical role of DNMTs or TET dioxygenases in normal embryonic development was demonstrated by several research studies [92,93]. Embryonic deletion of DNMT3 in neomycin conditional knockout mice results in neuronal dysfunction, associated with hypoactivity, motor abnormalities, fewer motor neurons, and a shortened lifespan [92]. Another study demonstrated that depletion of all forms of TETs in Tet triple-knockout mice arrests cell lineage differentiation and results in abnormal embryonic development [93].

The investigations of biopsies obtained from specific organs of human embryos demonstrated that the DNA methylation trajectory in the embryo undergoes considerable remodeling and modulations during the first to the second trimester of gestation (9, 18, and 22 weeks) [6,94]. In addition, DNMTs and TET enzymes are spatially and temporally expressed during neurogenesis in the developing fetus [95]. However, the tissue-specific methylation trajectory also continues during the early postnatal period [8]. Therefore, these “critical” periods of epigenetic remodeling are extremely sensitive to environmental stimuli, leading to stable large-scale changes in the embryo’s and postpartum baby’s tissues.

There is a scarcity of data regarding the effects of SAMe on embryonic epigenetic remodeling. Several studies reported the effects of methionine supplementation or diet with methionine restriction that allow extrapolation of their finding to SAMe’s effects, due to the conversion of methionine to SAMe by MAT enzymes. A deficiency of methionine, the precursor of SAMe, in the maternal diet causes abnormal cell metabolism through DNA hypomethylation [87]. Peñagaricano et al. [96] studied the effects of methionine supplementation on Holstein cow dams for 70 days during follicular development and early embryo development until day 7 post fertilization (Table 2). The addition of methionine to pregnant Holstein cows’ diet caused a significant decrease in the expression of most genes in the preimplantation embryo, consistent with reduced transcription of genes and increased methylation of specific genes [96]. Another study demonstrated that the addition of SAMe to in vitro cultured bovine embryos from the eight-cell to the blastocyst stage caused genome-wide hypermethylation mainly in exomic regions and the CpG islands. The analysis of differentially expressed genes revealed an association with the response to nutrients and developmental processes but not with the changes in methylated regions [97].

It was demonstrated that changes in the DNA methylation state during gestation, triggered by methyl donors, could exhibit a stable phenotypic change in offspring using the viable yellow agouti (A(vy)) mouse model [98,99,100]. This murine agouti gene A(vy) allelic expression is dependent on the methylation state of a transposon and of a promoter region of the gene, providing researchers with an excellent animal model for exploring the effect of chemical agents on epigenetic processes during gestation [101,102,103].

Cooney et al. [98] investigated the effects of different amounts of maternal dietary methyl supplements using betaine, choline, folic acid, B12 with or without methionine, and zinc during gestation on DNA methylation and offspring’s phenotype. They studied two inbred mouse strains that carried mutant alleles at the agouti locus. All these methyl donors are involved in one-carbon metabolism and regulate the synthesis of SAMe, and thereby could modulate the DNA methylation state. The methyl-donor in the maternal diet changed the offspring DNA methylome, changing the yellow coat color distribution to a pseudo agouti coat as a result of alterations in the gene expression.

SAMe, given orally to ICR mice on days 12–15 of gestation, resulted in significant gender-related changes in the expression of many genes in the brain of 1-day-old offspring [104]. The most prominent changes in gene expression were Vegfa and its receptor Flt1 (Fms-related receptor tyrosine kinase 1), which demonstrated upregulation by 390% and 219% in males, and 272% and 292% in females, respectively [105]. A single dose of VPA administered to pregnant mice on day 12 of gestation almost completely reversed the effects of SAMe, and the expression of all except 4 genes was similar to controls [104].

SAMe’s modulatory effects during gestation were also demonstrated by us in submissive (Sub) mice, a selectively inbred model of depression based on recurrent breeding using a social interaction and food competition paradigm [106,107]. The offspring of Sub mice that were treated by oral gavage with SAMe (30 mg/kg) on gestational days 12–14 demonstrated improved depression-like behavior at adulthood, especially in the Three Chamber test for sociability. This normalized social behavior was well correlated with Vegfa and its receptor Flt1 gene expression in the prefrontal cortex [108].

### 3.3. SAMe and Its Possible Effects on the Pregnant Mother and Offspring

Few studies have investigated the effects of SAMe treatment during pregnancy on the developing human embryo and fetus. Several clinical reports on the use of SAMe for the treatment of cholestasis in pregnancy demonstrated an absence of adverse effects for mothers or their infants [51,109,110]. Generally, the conducted clinical studies using SAMe in the pregnant population for the treatment of cholestasis support SAMe’s safety for both the mother and the fetus [111].

SAMe’s safety was examined in rats and New Zealand White rabbits [112] following intravenous and subcutaneous premating treatment or treatment during pregnancy and also during the peri- and post-natal periods. SAMe treatment was found to be safe, without adverse effects on either F0 or on the untreated F1 generations. In the treated dams, local tissue reaction at the injection sites and retardation of body weight gain were noted for the 400 mg/kg/day subcutaneous SAMe treatment regimen. In the intravenous studies, some rigidity and dyspnea were noted following administration. Following subcutaneous premating treatment, there was also evidence of histopathological changes in the kidneys of the female rats. However, in our studies [18,104], we did not observe any abnormal reactions of pregnant DAMs after oral SAMe administration.

### 3.4. In Summary

SAMe is an FDA-approved dietary supplement and is also approved for the treatment of major depressive disorder (MDD), osteoarthritis, and alcohol liver disease. SAMe facilitates DNA methylation by activating DNA methyltransferases that move the methyl group from SAMe to the 5′-position of a CpG. However, SAMe administration to pregnant women is restricted due to the lack of efficacy and safety data on the mother and embryo. Several animal studies have assessed the possible modulatory effect of SAMe during gestation on DNA methylation and gene expression and described the alleviation of several “epigenetic” neurobehavioral disorders, such as ASD and MDD, making SAMe a possible candidate for “epigenetic therapy”. The existing data on the possible damaging effects of SAMe on pregnancy outcomes are negative, but these studies were carried out in the last trimester of pregnancy. There are no studies on treatment during earlier phases when the embryo and fetus may be more susceptible to possible damage. More human studies are needed in order to investigate the possible efficacy of SAMe for the treatment of “epigenetic diseases” and the mechanism(s) of its interaction with VPA.

## 4. Choline and Pregnancy

Choline is an essential micronutrient used in various metabolic and physiologic reactions and serves as the starting compound for the biosynthesis of several important metabolites. Although choline is normally synthesized in the body, the majority comes from dietary sources, both animals and plants, because the choline formed in the body is in insufficient amounts to meet all metabolic demands [113,114].

During prenatal development, the demand for choline is especially high. Choline is involved in several critical processes of fetal development. During gestation, choline is involved in maternal and fetal tissue expansion [115]. It provides a substrate for the synthesis of phosphatidylcholine and sphingomyelin, major constituents of all cell membranes, and is required for brain development and fetal growth [115].

Choline also acts as a principal source of methyl groups (Figure 1). In the one-carbon cycle, choline’s oxidative derivative, betaine, converts homocysteine to methionine, which is used to generate the primary methyl donor S-adenosylmethionine (SAMe). Hence, choline can exert lasting effects on gene expression via epigenetic mechanisms [114].

Choline is the precursor of acetylcholine and is therefore especially needed for fetal brain development. Choline is also involved in the regulation of neuronal proliferation, differentiation, maturation, and regulation of gene expression and neuronal survival [116,117]. Choline has a significant impact on brain development and cognition and may, therefore, represent a potential intervention for cognitive impairments [118].

Choline participates in membrane formation and lipid membrane integrity. In addition, choline is an enzymatic co-factor involved in the conversion of homocysteine to methionine from 5-methyltetrahydrofolate-dependent remethylation of the homocysteine pathway in the one-carbon cycle via its metabolite trimethylglycine (betaine) [105,119]. Choline is actively absorbed from the blood by a number of choline-like transporters that also facilitates choline uptake into the mitochondria, where choline is transformed to betaine by oxidation [120]. Methionine adenosyltransferase knockout mice, which have impaired formation of SAMe, activate the gene expressing betaine-homocysteine methyltransferase. They therefore have increased dietary choline requirements [121].

### 4.1. The Impact of Choline on Pregnancy and Offspring in Rodents

Prenatal choline deficiency in rodents is associated with changes in specific gene and protein expression [122,123] and in global DNA methylation, especially in the brain [124]. Many studies have assessed the possible neurobehavioral effects of high doses of choline during pregnancy in mice and rats. For example, Kwan et al. [125] studied the effects of maternal choline supplementation on epigenetic markers in the mouse placenta. Exposure to high choline levels (5.6 g choline chloride/kg diet) during gestation altered the expression of several imprinted genes in a sex-specific manner. Choline supplementation also improved placental vascularization and perfusion [126].

Prenatal exposure to high doses of choline in rats resulted in improvement of cognitive function, spatial memory, and attentional function in offspring [127]. Treatment of pregnant rats with 25 mM choline via drinking water from days 11–18 of pregnancy resulted in a precocious capacity for spatial navigation, whereas control rats required 3 additional days of maturation to acquire this skill. Similar results were reported by Meck et al. [128]. In contrast, mice offspring of dams that received a low-choline diet during gestation showed disrupted retinal development and visual function [129].

### 4.2. Choline and Prenatal Exposure to Alcohol: Experiments in Animals

Fetal alcohol spectrum disorder (FASD) is considered one of the major public health challenges, with an incidence range of 0.8–5% in different countries [130,131]. Children prenatally exposed to alcohol exhibit growth retardation, facial malformations, and central nervous system injuries, and mental retardation and cognitive deficits, such as intellectual impairments, visual-spatial learning deficits, and verbal and non-verbal memory deficits [131]. The neurobehavioral impairments found in children with FASD last into adulthood, suggesting, in addition to the well-known damage of various brain circuits, the involvement of epigenetic patterns in the persistent programming effects of fetal alcohol exposure. Furthermore, prenatal alcohol exposure is associated with distinct DNA methylation patterns in children and adolescents [132], indicating the involvement of epigenetic mechanisms in mediating the range of symptoms observed in children with FASD.

Because choline is an essential nutrient that directly affects brain development and cognition [118], choline supplementation during pregnancy or the early postnatal period may be a potential intervention for the cognitive and other impairments associated with FASD.

Indeed, choline supplementation to animals exposed during pregnancy to high doses of ethanol generally resulted in alleviation of the damage induced by alcohol administration. For example, Steane et al. [133] treated ethanol-exposed pregnant rats with choline-supplemented chow (7.2 g choline/kg chow). Choline supplementation resulted in increased fetal weight by late gestation, ameliorating the deficits caused by ethanol consumption (Table 3).

In a sheep model of first-trimester binge alcohol drinking, choline (10 mg/kg in the daily food ration) administration from gestational day 4 until term mitigated the adverse neurobehavioral effects of alcohol binge drinking and additionally improved fetal appendicular bone length (femur and humerus) [134].

### 4.3. Human Studies

In humans, choline supplementation during pregnancy mitigated the adverse effects of heavy prenatal alcohol exposure on eyeblink conditioning, postnatal growth, and cognition as studied at 6.5 and 12 months of age [135]. Choline supplementation during pregnancy also mitigated prenatal alcohol exposure-related brain volume reductions and improved recognition memory at 12 months [136].

Kable et al. [137] followed pregnant women with reported alcohol use during pregnancy. Half of the women enrolled were assigned to receive daily prenatal vitamin/mineral supplement and the other half received a daily dose of vitamin/mineral supplement combined with choline (750 mg). Vitamin/mineral combined with choline supplementation was found to significantly improve basic attentional regulation systems, such as neurophysiological encoding and memory of visual stimuli, in the first year of life. This improvement was observed in both alcohol-exposed pregnancies and non- or low alcohol-exposed pregnancies (Table 4 and Table 5).

### 4.4. Human Studies Assessing the Possible Effects of Gestational Choline on Offspring in Normal Pregnancies and in Pregnancies Following High Alcohol Ingestion

Several human studies have examined the association between maternal choline intake or blood levels during pregnancy and cognitive development among their children. Contrary to the predictions from animal studies, not all human studies reported positive effects of maternal choline supplementation (Table 4 and Table 5).

Positive studies: In a randomized placebo-controlled clinical trial of dietary phosphatidylcholine supplementation by Ross et al. [138], the authors treated 50 healthy pregnant women with 900 mg choline/day, and 50 pregnant women with placebo, starting in the second trimester. They reported that at 5 weeks of age, infants treated with choline were significantly more likely to have normal cerebral inhibition in an auditory evoked-response task, a characteristic associated with a reduced risk of both attentional dysfunction and schizophrenia. 

In a prospective cohort study by Boeke et al. [139], the authors estimated the associations between intakes of choline or other nutrients, such as vitamin B12, betaine, and folate, during the first and second trimesters of pregnancy and offspring visual memory at 7 years of age. Maternal gestational choline intake (but not other nutrients), especially during the second trimester of pregnancy, was positively associated with children’s visual memory.

Caudill et al. [140] examined the effects of maternal choline supplementation (480 or 930 mg/ day) during the third trimester of pregnancy on infant’s cognition at 4, 7, 10, and 13 months of age. They found faster information processing speed in infants during 4–13 months in the 930 (*n* = 12) versus 480 mg/day group (*n* = 12).

Freedman et al. [141] compared serum-free choline and betaine concentrations in 162 mothers with different infections during week 16 of gestation. They found significantly improved development of newborns’ cerebral inhibition and behavioral regulation in 1-year-old infants born to infected mothers with higher gestational serum choline concentrations. Higher maternal plasma choline at 16 weeks of gestation was also associated with higher processing speed and decreased problems regarding social withdrawal among the children at 4 years of age (Table 4).

In contrast to these findings, Cheatham et al. [142] treated pregnant women with supplemental phosphatidylcholine (750 mg choline/day) or placebo from 18 weeks of gestation until postnatal day 90. Their infants were tested for short-term visuospatial memory, long-term episodic memory, language development, and global development at 10 and 12 months of age. The authors did not find any effect of maternal or postnatal choline supplementation on infants’ brain function.

### 4.5. Early Postnatal Choline Treatment of Children with FASD

Several studies have evaluated the effects of choline supplementation on young children prenatally exposed to alcohol. The results are controversial (Table 5).

Positive results were reported by Wozniac et al. [143] in a cohort of 51 preschool-aged children with FASD treated daily with 500 mg choline or with placebo for 9 months. Choline supplementation improved the memory function in children between the ages of 2.5 and 5 years. Later, at a mean age of 8.5 years, 31 of the children (16 placebo, 15 choline) underwent measurements of their intelligence, memory, executive functioning, and behavior [16]. Children who received choline had higher non-verbal intelligence, higher visual-spatial skills, higher working memory ability, better verbal memory, and fewer behavioral symptoms of attention deficit hyperactivity disorder than the placebo group. In the same group of children with FASD, choline supplementation also reduced DNA methylation and increased the expression of stress regulatory genes (PER2 and POMC) [144], emphasizing the epigenetic influence of choline.

In contrast to these positive findings, Nguyen et al. [145] studied 55 children aged 5–10 years that were exposed prenatally to heavy maternal alcohol drinking. They received either 625 mg choline/day for 6 weeks (*n* = 29) or placebo (*n* = 26). The children were administered a standardized neuropsychological test battery before and immediately after the intervention. The authors reported that choline-treated children did not differentially improve in cognitive performance in any domain, including learning and memory, executive function, and sustained attention. The negative results could be explained by the short treatment duration. More studies are needed to properly evaluate the possible effects of choline treatment on children with FASD.

### 4.6. In Summary

Positive effects of prenatal choline supplementation were described in several studies, indicating the possible benefits of choline dietary intake during pregnancy in regulating the cognitive functions of offspring, especially in children with FASD. There seems to be no damaging effects of choline on the course of pregnancy or on the conceptus. Because of the limited number of studies that have explored the effects of prenatal and postnatal dietary choline interventions and their controversial results, more research is needed to reach conclusions about its effectiveness and to find the optimal supplementation dosage and periods. Once the data are clear, it will be possible to better judge the efficacy of choline in pregnancy for possible “epigenetic therapy”.

## 5. Discussion and Conclusions

We described the main effects of three agents that have the ability to induce epigenetic modifications: valproic acid, a well-established human teratogen that is contraindicated in pregnancy unless doses lower than 600 mg/day are used; S-adenosylmethionine (SAMe); and choline. These substances are known for their involvement in the processes of gene activation and silencing as they are important components of one carbon metabolism (SAMe and choline) and histone functions (VPA). SAMe and VPA seem to have antagonistic effects on gene expression, as evidenced in animal experiments. Additional important substances involved in the one carbon cycle are methionine and folic acid, which may also serve as “epigenetic drugs”, but they are not discussed in this review.

SAMe, a recognized food additive, has been successfully used in the last years as an adjunct therapy for several psychiatric disorders and choline is a well-recognized food additive. However, we are still far away from their use as epigenetic modulators or their regular use in medical practice. The daily adult dose of SAMe is 400–800 mg, that of choline is 500–1000 mg, and the daily dose of VPA in pregnancy should not exceed 600 mg due to its high teratogenicity. Studies have shown that each of these substances is able to alleviate the severity of neurobehavioral symptoms in animal models of diseases, such as ASD, MDD, MTHFR deficiency, Rett syndrome, and alcoholic embryopathy, by inducing changes in the expression of affected genes or by other, yet unknown, mechanisms. However, we are still missing clinical trials that might enable the translation of the beneficial effects of these substances to the treatment of human “epigenetic diseases”.

## Figures and Tables

**Table 1:** The effects of prenatal or early postnatal VPA administration to alleviate the symptoms of several epigenetic diseases.

First Editor	Animals	VPA Treatment	Outcomes
Roy et al., 2008	Mice with reduced activity of Mthfr gene (Mthfr+/−)	Single dose of 300 mg/kg VPA on day 8.5 of pregnancy	Increase in the expression of Mthfr gene and normalization of MTHFR protein; improved fetal outcome compared to treated normal mice
Guo et al., 2014	Six-week-old mice with MECP2 deficiency (Rett syndrome)	Daily injections of 350 mg/kg VPA for 2 weeks	Enhanced expression of MeCP2 gene and increased MeCP2 protein with improvement in the clinical symptoms of Rett syndrome
Cohen et al., 2013	Normal rats	350 mg/kg VPA on day 13 of gestation in normal rats	Increased expression in the brain of genes encoding for acetylation-sensitive proteins and increased social investigation and play fighting
Jacob et al., 2014	Zebrafish	0.625 mM VPA in water	Downregulation of the proneuronal gene ascl1b and inhibition of serotonin synthesis in the brain
Ornoy et al., 2019	4-day-old ICR mice	300 mg/kg VPA injected to 4-day-old offspring or VPA and SAMe 30 mg/kg during postnatal days 4–6	Induction of autistic-like behavior and increased oxidative stress in the prefrontal cortex ameliorated by SAMe
Weinstein et al., 2019	4-day-old ICR mice	300 mg/kg VPA injected to 4-day-old offspring or VPA and SAMe 30 mg/kg during postnatal days 4–6	VPA induced changes in the expression of neurophysiologic and neuropathlogic genes in the prefrontal cortex that were reversed to normal by SAMe

**Table 2:** The effects of prenatal SAMe or methionine administration in pregnant animals or human.

First Editor	Animal/Human	Treatment	Outcomes
Peñagaricano et al., 2013	Holstein cows early embryo	Methionine supplementation during follicular phase and early embryo development, until day 7.	In total, 276 of the 10,662 genes analyzed showed significant differences following treatment. Maternal methionine supplementation resulted in decreased expression of most genes, reduced transcription, and increased methylation of specific genes.
Shojaei Saadi et al., 2002	In vitro cultured bovine embryos	SAMe treatment from the eight-cell stage to the blastocyst stage.	SAMe induced genome-wide hypermethylation mainly in exonic regions and in CpG islands. Differentially expressed genes were associated with the response to nutrients and developmental processes.
Cooney et al., 2002	Viable yellow agouti (A(vy)) mouse strains that carry mutant alleles at the agouti locus	Methyl donor diets: betaine, choline, folic acid, B12 with or without methionine, and zinc during gestation.	The increase in offspring DNA methylation state resulted in a change from a yellow coat color distribution to a pseudo agouti coat as a result of alterations in the gene expression.
Weinstein et al., 2020	ICR mice	Oral SAMe given to DAMs on days 12–15 of gestation.	Significant gender-related changes in the expression of many genes in the brain of 1-day-old offspring. The most prominent changes in gene expression were Vegfa and its receptor Flt1.
Becker et al., 2021	Sub mice derived from Sabra	Oral gavage with SAMe (20 mg/kg) on gestational days 12–14.	Improved depression-like behavior at adulthood, especially in the three chamber test for sociability. Increased expression of Vegfa and its receptor Flt1 genes in the prefrontal cortex at 90 days of age.
Tomáš Binder et al., 1990	Pregnant Women with intrahepatic cholestasis of pregnancy (ICP)	SAMe for the treatment of intrahepatic cholestasis.	No adverse effects were noted on the fetuses or neonates.
Frezza, M et al., 1990	Thirty patients in the last trimester of pregnancy with intrahepatic cholestasis of pregnancy (ICP)	SAMe (800 mg/day i.v.) or placebo until delivery for a mean period of 18 days.	No adverse reactions on mother or child were recorded during SAMe treatment, and at follow-up of the children. Possibly decreased rate of prematurity compared to placebo treated.
Coltorti et al., 1990	Eighteen patients with intrahepatic cholestasis of pregnancy (ICP)	SAMe 900 mg/day or placebo was administered by daily intravenous infusions for 20 days.	No relevant adverse reactions were detected. All newborns had Apgar scores greater than 7 and normal postnatal development.

**Table 3:** The effects of prenatal choline administration on the neurodevelopment of rats and mice with or without exposure to prenatal ethanol (alcohol).

First Editor	Rodents	Treatment	Outcome
Albright et al., 1999	Sprague-Dawley rats fetal brain sections were collected on days 18 and 20 of pregnancy	Varying levels of dietary choline for 6 days from gestational day 12.	Choline deficiency-related brain changes including an increased number of apoptotic cells in the dentate gyrus and increased expression of TOAD-64, a neuronal differentiation marker, in the hippocampus.
Albright et al., 2001	Sprague-Dawley rats fetal brain sections were collected on days 18 and 20 of pregnancy	Varying levels of dietary choline for 6 days from gestational day 12.	Maternal dietary choline deficiency changed the localization of p15Ink4B and p27Kip1 cyclin-dependent kinase inhibitors in the offspring hippocampus.
Mellott et al., 2004	Sprague-Dawley rats	Pregnant rats fed a choline-supplemented diet for 8 days (between embryonic days 11 and 18)	Choline-supplemented rats showed evidence of a precocious capacity for the spatial navigation water maze task. Choline also in creased activation of mitogen-activated protein kinase (MAPK) and cAMP-response element binding protein (CREB) in the hippocampus.
Niculescu et al., 2006	C57 BL/6 mice	Pregnant dams fed deficient or normal in choline content diet from days 12 to 17 of pregnancy. Fetal brains collected on embryonic day 17.	Choline deficiency increased protein levels of kinase-associated phosphatase (Kap) and p15INK4b (two cell cycle inhibitors) and decreased gene-specific DNA methylation in the offspring brain.
Cheng et al., 2008	Sprague-Dawley rats	Pregnant rats fed normal choline (1.1 g/kg) or supplemental choline (5.0 g/kg) during embryonic days 12–17. Male and female offspring conducted behavioral training at 7 months of age.	Prenatal choline supplement-action was associated with an improvement of cognitive function, spatial memory, and attentional function
Kwan et al., 2018	Swiss Albino mice	Pregnant mice fed a 1X (1.4 g choline chloride/kg diet) or 4X choline (5.6 g choline chloride/kg diet) diet from embryonic day 0.5. placentas collected on embryonic day 15.5.	High choline levels during gestation altered the expression of several imprinted genes in a sex-specific manner.
Trujillo-Gonzalez et al., 2019	*NestinCFPnuc Nestin-CreER^T2^, Ai9* and C57BL/6J mice	Pregnant dams randomly assigned to either adequate (1.4 g/kg of choline chloride) or low-choline diet (1.2 g/kg of choline chloride) administered starting at day 11.5 of pregnancy.	Low-choline diet during gestation was associated with disrupted retina development and visual function.
Steane et al., 2021	Sprague Dawley rats	Female rats exposed to 12.5% ethanol from 4 days prior to 4 days after conception. From day 5 of pregnancy, dams were placed on different choline levels chow (1.6–7.2 g choline/kg chow). Fetuses and placentas were collected on day 20 of pregnancy for analysis.	Choline supplementation resulted in increased fetal weight by late gestation, ameliorating the deficits caused by maternal ethanol consumption

**Table 4:** The effects of prenatal choline administration on the neurodevelopment of children with FASD.

First Editor	Participants	Treatment	Outcomes
Ross et al., 2013	100 healthy pregnant women and their infants.	Phospohadtidycholine (approximately 900 mg choline/day) from the second trimester of pregnancy and 100 mg until the age of 3 months.	Infants treated with choline are significantly more likely to have normal cerebral inhibition at 5 weeks of age.
Kable et al., 2015	Pregnant women with moderate/heavy alcohol use.	750 mg choline (*n* = 37) multivitamin/mineral supplement (*n* = 23); combination of both treatments (*n* = 19), standard care (*n* = 35)	Multivitamin/mineral combined with choline supplementation significantly improved basic attentional regulation systems.
Jacobson et al., 2018	69 pregnant heavy drinker mothers and their infants.	2000 mg choline (*n* = 34 or placebo (*n* = 35) from mid-pregnancy until delivery	Choline treatment improved weight, postnatal growth, cognition, and eye-blink conditioning at 6.5 and 12 months of age.
Caudil et al., 2018	26 pregnant women entering their third trimester until delivery, and their infants	Either 480 mg choline/d (*n* = 13) or 930 mg choline/d (*n* = 13).	Higher choline levels associated with faster information processing speed in infants at 4–13 months.
Freedman et al., 2019	162 Pregnant women with different infections at 16 week of gestation and their infants at 1 year of age	Maternal serum choline and betaine levels were measured at 16 week of gestation.	Higher gestational choline concentrations were associated with improved development of cerebral inhibition and cerebral regulation at the age of one year.
Warton et al., 2021	52 mothers with heavy drinking and their infants.	2000 mg choline (*n* = 28) or placebo (*n* = 24) from mid-pregnancy until delivery.	Choline supplementation during pregnancy mitigated regional volume reductions in alcohol-exposed infants, with larger volumes associated with improved 12-month recognition memory.
Hunter et al., 2021	122 pregnant mothers and their children from 3 months of age; 48 children completed the assessment at 4 years of age.	Maternal serum choline and betaine levels were measured at 16 and 28 weeks of gestation.	Prenatal maternal choline levels were positively associated with higher processing speed and decreased problems in social withdrawal.
Cheatham et al., 2012	140 pregnant women assigned from 18 weeks of gestation, and their infants	750 mg phosphatidyl-choline (*n* = 49) or placebo (*n* = 50) daily from 18 weeks of gestation until 90 days post-partum and their infants	No significant effect of phosphatidylcholine supplement detected on short-term visuospatial memory, long-term episodic memory, language development and global development at 10 and 12 months of age.

**Table 5:** The effects of early postnatal choline administration on the neurodevelopment of children with FASD.

First Editor	Participants	Treatment	Outcomes
Wozniak et al., 2015	60 children aged 2.5–5 years at enrollment, with FASDs	500 mg choline or placebo daily for 9 months	Choline significantly improved delayed sequential memory in 2–3-year-olds.
Sarkar et al., 2019	60 children aged 2.5–5 years at enrollment, with FASDs	500 mg choline or a placebo daily for 9 months	Choline supplementation reduced DNA methylation of hPER2 and hPOMC genes and increased the expression of stress regulatory genes.
Wozniak et al., 2020	Follow up of 31 children with FASDs mean age 8.6 years	500 mg choline (*n* = 15) or a placebo (*n* = 16) daily for 9 months between 2.5 and 5 years	Choline significantly improved nonverbal intelligence, higher visual-spatial skills, working memory ability, and verbal memory, and decreased behavioral symptoms of attention deficit hyperactivity disorder.
Nguyen et al., 2016	55 children aged 5–10 years, with confirmed histories of heavy prenatal alcohol exposure.	625 mg choline (*n* = 29) or placebo (*n* = 26) daily for 6 weeks	Choline supplementation did not improve cognitive performance in any domain.

## Data Availability

Data sharing not applicable.

## Abbreviations

A(vy)	viable yellow agouti mice
ASD	Autism spectrum disorder
DNMTS	DNA methyltransferases
FASD	Fetal alcohol spectrum disorder
Flt1	Fms related receptor tyrosine kinase 1
GSH	Hepatic glutathione
HCC	Hepatocellular carcinoma
HDAC	Histone deacetylases
ICM	Inner cell mass
MAT	Methionine adenosyltransferase
MDD	Major depressive disorder
MTHFR	Methylenetetrahydrofolate reductase
SAMe	S-adenosylmethionine
Sub	Submissive mice
TET	Ten–eleven translocation dioxygenases
VEGF	Vascular endothelial growth factor
VPA	Valproic acid
